# The Validity of the Session Rating of Perceived Exertion Method for Measuring Internal Training Load in Professional Classical Ballet Dancers

**DOI:** 10.3389/fphys.2020.00480

**Published:** 2020-05-14

**Authors:** Joseph W. Shaw, Matthew Springham, Derrick D. Brown, Adam M. Mattiussi, Charles R. Pedlar, Jamie Tallent

**Affiliations:** ^1^Faculty of Sport, Health and Applied Science, St Mary’s University, Twickenham, United Kingdom; ^2^Ballet Healthcare, The Royal Ballet, Royal Opera House, London, United Kingdom; ^3^Institute of Sport Science, Dance Science, University of Bern, Bern, Switzerland; ^4^Division of Surgery and Interventional Science, University College London, London, United Kingdom

**Keywords:** RPE, training load, dance, athlete monitoring, athlete wellness, ballet

## Abstract

The aim of this study was to investigate the convergent validity of session rating of perceived exertion (s-RPE) with objective measures of internal training load (TL) in professional classical ballet dancers. Heart rate and s-RPE data were collected in 22 professional classical ballet dancers across a total of 218 ballet class or rehearsal sessions. Eleven participants completed at least 9 sessions, and were therefore included in analyses of individual relationships between s-RPE and objective measures. To calculate s-RPE, the session duration was multiplied by the RPE, measured using the modified Borg CR-10 scale. The Edwards summated heart rate zones (Edwards TRIMP) and Banister training impulse (Banister TRIMP) methods were used as criterion measures of internal TL. Pearson product-moment correlation coefficients were used to determine intra-individual relationships between s-RPE and objective measures. Repeated measures correlations were used to identify intra-individual relationships common across the cohort. Positive linear relationships were seen between s-RPE and objective measures across all session types [Edwards TRIMP: *r*_rm (195)_ = 0.81, *p* < 0.001; Banister TRIMP: *r*_rm (195)_ = 0.79, *p* < 0.001], in ballet class [Edwards TRIMP: *r*_rm (58)_ = 0.64, *p* < 0.001; Banister TRIMP: *r*_rm (58)_ = 0.59, *p* < 0.001], and in rehearsals [Edwards TRIMP: *r*_rm (119)_ = 0.82, *p* < 0.001; Banister TRIMP: *r*_rm (119)_ = 0.80, *p* < 0.001], as well as across both males [Edwards TRIMP: *r*_rm (136)_ = 0.82, *p* < 0.001; Banister TRIMP: *r*_rm (136)_ = 0.80, *p* < 0.001], and females [Edwards TRIMP: *r*_rm (57)_ = 0.80, *p* < 0.001; Banister TRIMP: *r*_rm (57)_ = 0.78, *p* < 0.001]. Intra-individual correlation coefficients ranged from 0.46–0.96 [Edwards TRIMP: mean *r* = 0.81 ± 0.11, *p* = 0.051 – < 0.001; Banister TRIMP: mean *r* = 0.78 ± 0.14, *p* = 0.13– < 0.001]. These results demonstrate that s-RPE is a valid and practical method for measuring internal TL in professional classical ballet dancers.

## Introduction

Classical ballet is an intermittent activity, consisting of high intensity explosive actions interspersed with periods of lower intensity technical movements or inactivity (Wyon et al., 2011). Each season, a professional ballet company may perform as many as 145 shows of 15 different productions (Allen et al., 2012). To prepare for the performance schedule, professional dancers will typically complete 1.5 h of ballet class, and between 2 and 7 h of rehearsals each working day (Cohen et al., 1980). The resulting training volume is greater than values previously reported in elite team sport (Ekstrand et al., 2011) and endurance athletes (Mårtensson et al., 2014), and has been linked with overtraining and injury (Wyon, 2010). The periodization of training load (TL) has been proposed as a strategy to optimize performance and reduce the risk of overuse injury within dance populations (Wyon, 2010).

Training load can be described in terms of the physical work performed during exercise, and/or the psychophysiological responses to that work, i.e., the external and internal TL, respectively (Impellizzeri et al., 2019). It is the internal TL, however, which provides the stimulus for physiological adaptation. A valid measure of internal TL is therefore essential for the effective periodization of a rehearsal schedule within a professional ballet company. Dance intensity during ballet rehearsal and performance has previously been measured using oxygen uptake, heart rate (HR), and blood lactate concentration (Schantz and Astrand, 1984). Given the number of dancers employed by professional companies and the esthetic demands of ballet performance, these solutions are impractical for daily monitoring. The session rating of perceived exertion (s-RPE) method, derived from the product of session duration, and RPE, has therefore been used as a time-sensitive and cost-effective method of quantifying internal TL in dance populations (Da Silva et al., 2015). Simple derivatives such as monotony and strain may subsequently provide practitioners with insights into maladaptive responses to training such as overtraining and illness. The s-RPE method is therefore commonly used in both research and applied practice, and has been validated across a range of modalities including team (Impellizzeri et al., 2004), combat (Padulo et al., 2014), and endurance sports (DellaValle and Haas, 2013). Although the validity of s-RPE has been demonstrated in populations of contemporary (Jeffries et al., 2016; Surgenor and Wyon, 2019) and step dancers (Ozkan and Kin-Isler, 2007), to our knowledge it has not been investigated within ballet dancers.

Jeffries et al. (2016) investigated relationships between s-RPE and objective measures of internal TL in contemporary dancers during contemporary class, contemporary rehearsal, and ballet class. Group correlations ranging from 0.44–0.73 and 0.52–0.72 were seen for contemporary class and rehearsal, respectively, while relationships were weaker in ballet class (*r* = 0.32–0.58). Similarly, in a cohort of pre-professional contemporary dancers, Surgenor and Wyon (2019) saw a strong group correlation (*r* = 0.72) between contemporary class and rehearsals, but a moderate relationship (*r* = 0.46) in ballet class alone. The weaker relationships seen in ballet class compared with rehearsals could have been because ballet was not the dancers’ primary genre, or because of a difference in the genres themselves. These distinctions between dance/genre specific sessions (i.e., ballet class vs. rehearsal) are consistent with research in sporting contexts (Lupo et al., 2017a, b). Furthermore, factors such as the athlete’s sex, age, and fitness level have all been suggested to influence the relationship between s-RPE and objective measures of TL (Haddad et al., 2017).

Investigations into the influence of sex on the perception of exercise have demonstrated mixed results. While no difference in RPE was seen between male and female college students during a graded exercise test (Kim and Lee, 2011), male and female champion cross country runners registered differing perceptions of “hard” sessions (Barnes, 2017). Within classical ballet, the roles and technical choreographies performed differ across sex. Male dancers are required to lift their partners, demanding significant full body strength and control (Wyon et al., 2011). Conversely, female dancers are required to dance en pointe, placing stress on the foot and ankle (Teitz et al., 1985). In this regard, male and female roles are comprised of sufficiently different demands to be considered separate modalities. It is therefore important to understand the extent to which s-RPE is a valid measure of internal TL in both male and female dancers.

The primary aim of this study was to investigate the construct validity of s-RPE as a measure of internal TL in professional classical ballet dancers, by examining the convergence with two validated TL measures derived from HR. The secondary aim was to understand the effect of session type and sex on this relationship.

## Materials and Methods

### Participants

A sample of 13 male (25.5 ± 5.3 years, 179.7 ± 4.0 cm, 73.2 ± 5.2 kg, and 7.8 ± 5.6 years professional) and 9 female (25.2 ± 4.4 years, 164.0 ± 3.3 cm, 52.9 ± 4.1 kg, and 7.4 ± 4.2 years professional) dancers from a professional ballet company volunteered to take part in the study. The sample consisted of dancers of the following ranks within the company hierarchy: one Apprentice, seven Artists, five First Artists, three Soloists, four First Soloists, and two Principal dancers. Apprentices, Artists, and First Artists make up the company’s Corps de Ballet, while Soloists, First Soloists, and Principal dancers perform increasingly featured roles. Prior to the onset of data collection, participants were given a full written explanation of the study aims and protocol and gave written informed consent. The protocol was approved by the St Mary’s University board of ethics in accordance with the Declaration of Helsinki.

### Experimental Design

A correlational study design was employed between April and October 2019. Participants were given the freedom to select the days during which data collection would take place. HR and s-RPE data were collected following the final session of each day. Edwards summated HR zones (Edwards TRIMP; Edwards, 1993) and Banister training impulse (Banister TRIMP; Banister, 1991) were calculated as criterion measures of internal TL, consistent with previous validations of s-RPE. It should therefore be noted that the criterions were primarily measures of aerobic demand, and not measures of other physiological demands (e.g., anaerobic, neuromuscular). Throughout the data collection period participants completed their normal rehearsal schedules as prescribed by the company’s artistic staff. For analyses of intra-individual relationships between measures, in order to achieve sufficient power to identify any convergence greater than *r* = 0.50, a sample of 23 sessions per participant was required (α = 0.05, β = 0.80, and *r* = 0.50). Collecting this volume of data was impractical in the present cohort; we therefore present intra-individual correlations where participants exceeded the required sample size for the expected correlation coefficient (α = 0.05, β = 0.80, *r* = 0.80, and *n* = 9), based on similar investigations (Lupo et al., 2014).

### Objective Measures of Internal Training Load

Heart rate data were collected during each session using a Polar H1 sensor (Polar Electro, Kempele, Finland) secured to the chest via an elastic strap and recorded by a wearable activity monitoring unit (ClearSky T6, Catapult Sports, Australia). Following the final session of each day, data were downloaded using Openfield Cloud Analytics software (Catapult Sports, Australia). Individual session data were then exported for external analysis. Maximum HR was calculated as the highest of either the age predicted maximum (Tanaka et al., 2001) or the peak value recorded during the data collection period. Edwards TRIMP was calculated by multiplying the time spent in five HR zones by a corresponding coefficient (50–60% HR_max_ = 1; 60–70% HR_max_ = 2; 70–80% HR_max_ = 3; 80–90% HR_max_ = 4; and 90–100% HR_max_ = 5), the results of which were then summed. Banister TRIMP was calculated using the equation:

$$B\,a\,n\,i\,s\,t\,e\,r\,T\,R\,I\,M\,P=D\times H\,R_{R}\times A\times e^{B\times H\,R_{\mathrm{R .}}}$$

where D = the session duration, A = 0.64 for men and 0.86 for women, B = 1.92 for men and 1.67 for women, and HR_*R*_ was calculated using the equation:

$$H\,R_{R}=\frac{H\,R_{e\,x}-H\,R_{r\,e\,s\,t}}{H\,R_{\text{max}}-H\,R_{r\,e\,s\,t}}$$

where HR_ex_ = mean HR during exercise, HR_rest_ = resting HR, and HR_max_ = maximal HR.

### Measurement of Session-RPE

For the measurement of s-RPE, the modified Borg CR-10 scale was used to quantify session intensity (Foster et al., 2001). Approximately 15 min following the final session of each day, the lead investigator met with each participant individually to record a RPE for each session that day. The use of retrospective RPEs was important to ensure the results were ecologically valid; within large ballet companies, dancers have multiple sessions per day, and may each be on a unique schedule, making it impractical to collect data after each session. Within sporting environments, the use of retrospective s-RPEs has been shown to be methodologically robust (Christen et al., 2016; Fanchini et al., 2016). The participant was shown the Borg CR-10 scale and asked about each session in the form of the question: “What was the intensity of your 1 pm rehearsal?” This differs from the original phrasing (“How was your workout?”), with the aim of directing the participant toward giving solely an intensity rating, and not a rating that takes session duration into account (Coutts et al., 2018). Participants were directed to first focus on a descriptive anchor, and then select a corresponding numerical value. If appropriate, participants were given the option to divide the session into multiple sections and give a separate RPE for each. The RPE was multiplied by the session duration (mins) to calculate s-RPE.

### Statistical Analysis

Intra-individual relationships were analyzed using Pearson’s product-moment correlation coefficients. This decision was made despite both s-RPE and objective measures of TL being non-normally distributed, as the rate type 1 error is relatively robust to non-normality when sample sizes are not especially small (Bishara and Hittner, 2012). This allowed for comparisons to be made with previous investigations into the validity of s-RPE in athletic populations, which have almost exclusively used Pearson’s r (Haddad et al., 2017). To investigate the common intra-individual relationships across the entire cohort, a repeated measures correlation (rmcorr; Bland and Altman, 1995) was conducted using the R package “rmcorr” (Bakdash and Marusich, 2017). Data were subsequently stratified, and rmcorr were conducted on sub-groups to compare relationships between sex (i.e., male and female sub-groups), and between session type (i.e., ballet class and rehearsal sub-groups). Statistical significance was set at *p* ≤ 0.05. In line with previous research (Jeffries et al., 2016), the magnitude of the correlation coefficient was interpreted as follows: <0.10 trivial, 0.10–0.29 small, 0.30–0.49 moderate, 0.50–0.69 large, 0.70–0.89 very large, and 0.90–1.0 almost perfect. All analyses were completed using R version 3.5.3.

## Results

**FIGURE 1:**
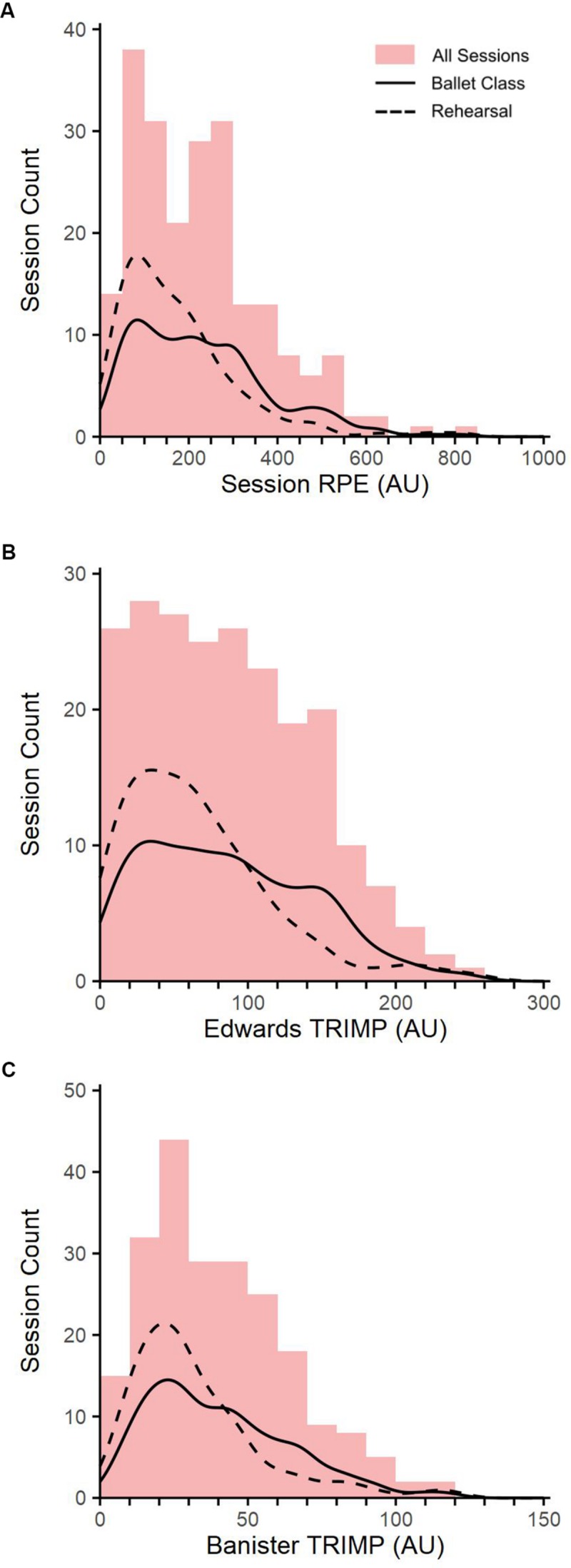
The distribution of **(A)** session rating of perceived exertion (Session RPE); **(B)** Edwards summated heart rate zones (Edwards TRIMP); and **(C)** Banister training impulse (Banister TRIMP) measures during all sessions (red bars), ballet class (solid line), and rehearsals (dashed line).

Data were collected across of 79 ballet classes and 139 rehearsals. Participants completed a mean of 9.9 ± 7.7 sessions each. Of the initial cohort of 22, 11 participants completed at least 9 sessions, and were therefore included in intra-individual analyses. One participant completed only one session, and therefore did not qualify for either intra-individual correlation, nor repeated measures correlation. The mean session duration (hh:mm:ss) was 01:12:10 ± 00:09:11, 00:52:31 ± 00:22:19, and 00:59:38 ± 00:20:53 for ballet class, rehearsals, and all sessions, respectively. Descriptive statistics for s-RPE, Edwards TRIMP, and Banister TRIMP are shown in Figure 1.

**FIGURE 2:**
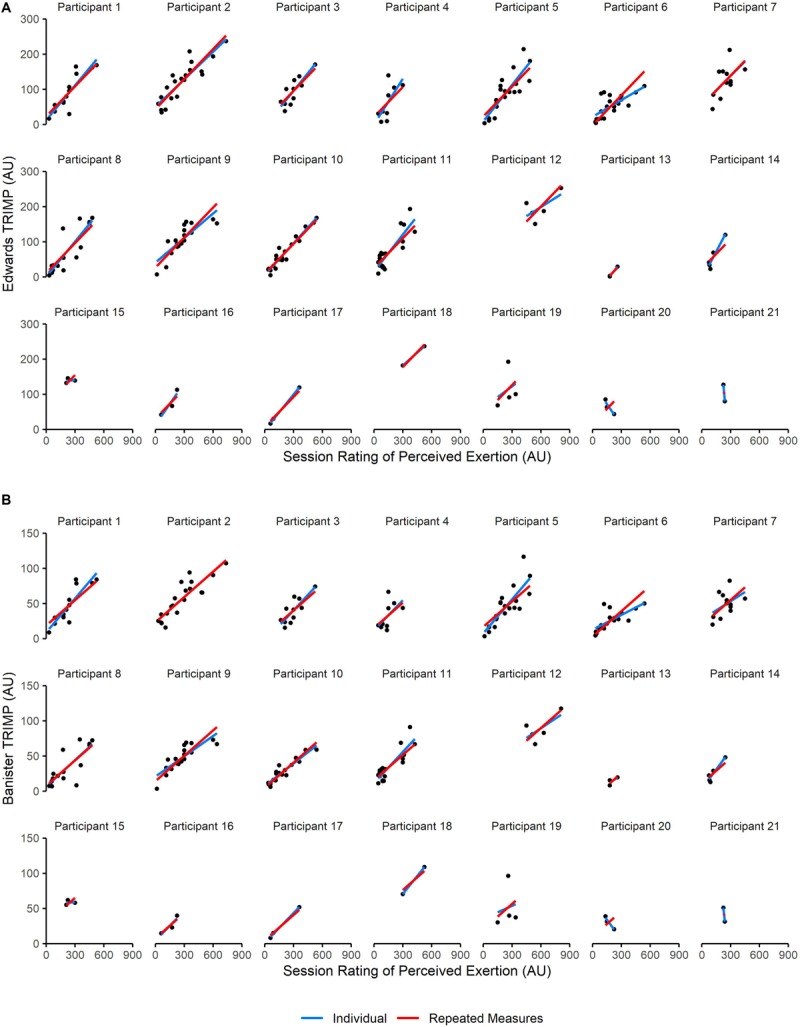
A comparison of Pearson’s correlation and repeated measures correlation (rmcorr) results for relationships between session rating of perceived exertion (session-RPE) and **(A)** Edwards summated heart rate zones method (Edwards TRIMP), and **(B)** Banister training impulse (Banister TRIMP). Each session completed by a participant is represented by a black circle. Blue lines depict individual Pearson’s correlations for each participant. Red lines depict the goodness of the rmcorr fit for each participant. Correlation coefficients, *p* values, and 95% confidence intervals for each participant who completed at least 9 sessions can be found in Table 1.

Repeated measures correlations revealed very large positive relationships between s-RPE and objective measures across all sessions [Edwards TRIMP: *r*_rm (195)_ = 0.81, *p* < 0.001, and 95% CI (0.76–0.86); Banister TRIMP: *r*_rm (195)_ = 0.79, *p* < 0.001, and 95% CI (0.73–0.84)]. Large [Edwards TRIMP: *r*_rm (58)_ = 0.64, *p* < 0.001, and 95% CI (0.45–0.77); Banister TRIMP: *r*_rm (58)_ = 0.59, *p* < 0.001, and 95% CI (0.39–0.74)] and very large [Edwards TRIMP: *r*_rm (119)_ = 0.82, *p* < 0.001, and 95% CI (0.75–0.87); Banister TRIMP: *r*_rm (119)_ = 0.80, *p* < 0.001, and 95% CI (0.72–0.85)] rmcorr were seen between s-RPE and Edwards TL in ballet class and rehearsals, respectively. Very large rmcorr between s-RPE and objective measures were seen for both male [Edwards TRIMP: *r*_rm (136)_ = 0.82, *p* < 0.001, and 95% CI (0.76–0.87); Banister TRIMP: *r*_rm (136)_ = 0.80, *p* < 0.001, and 95% CI (0.73–0.85)] and female [Edwards TRIMP: *r*_rm (57)_ = 0.80, *p* < 0.001, and 95% CI (0.68–0.88); Banister TRIMP: *r*_rm (57)_ = 0.78, *p* < 0.001, and 95% CI (0.66–0.87)] participants. Results of intra-individual correlations between s-RPE and objective measures are reported in Table 1. A comparison of the rmcorr and Pearson’s correlation results for each participant are shown in Figure 2.

**TABLE 1:** Correlation coefficients, *p* values, and 95% confidence intervals for intra-individual relationships between session rating of perceived exertion, and Edwards and Banister TRIMPs.

	**Sex**	**Sessions**	**Edwards TRIMP**			**Banister TRIMP**		
			***r***	***p***	**95% CI**	***r***	***p***	**95% CI**
P1	F	13	0.88	<0.001	0.64–0.96	0.88	<0.001	0.64–0.96
P2	F	20	0.88	<0.001	0.72–0.95	0.88	<0.001	0.72–0.95
P3	F	11	0.84	<0.001	0.48–0.96	0.81	<0.001	0.41–0.95
P4	F	9	0.68	0.045	0.03–0.93	0.56	0.113	-0.17–0.89
P5	M	21	0.85	<0.001	0.66–0.94	0.83	<0.001	0.62–0.93
P6	M	20	0.70	<0.001	0.37–0.87	0.73	<0.001	0.42–0.89
P7	M	12	0.57	0.051	-0.01–0.86	0.46	0.132	-0.15–0.82
P8	M	16	0.87	<0.001	0.66–0.95	0.80	<0.001	0.50–0.93
P8	M	20	0.83	<0.001	0.61–0.93	0.83	<0.001	0.61–0.93
P10	M	20	0.96	<0.001	0.90–0.98	0.95	<0.001	0.88–0.98
P11	M	22	0.86	<0.001	0.69–0.94	0.87	<0.001	0.71–0.94
Mean (± SD)	–	16.73 ± 4.5	0.81 ± 0.11	–	–	0.78 ± 0.14	–	–
Range	–	9–22	0.57–0.96	–	–	0.46–0.95	–	–

*Raw data for each participant can be seen in Figure 2. TRIMP, Training Impulse; P, Participant; F, Female; M, Male; and CI, Confidence Interval.*

## Discussion

To our knowledge, this is the first study to investigate the validity of s-RPE for the quantification of internal TL within a cohort of professional ballet dancers. The present results demonstrate large rmcorr between s-RPE and objective measures of TL, as well as intra-individual relationships ranging from moderate to almost perfect. Based on these results, the s-RPE method can be considered a valid measure of internal TL in professional classical ballet dancers.

Both the rmcorr and individual correlations observed between s-RPE and objective measures in the present study are slightly larger than both group (*r* = 0.71; Surgenor and Wyon, 2019) and individual (*r* = 0.72 ± 0.13; Jeffries et al., 2016) correlation coefficients reported in pre-professional contemporary dancers. Classical ballet and contemporary dance differ in their frequency of jumps (4.99 ± 4.93 vs. 1.71 ± 2.21 jumps per min), lifts (0.97 ± 2.53 vs. 0.12 ± 0.23 lifts per min), and changes of direction (3.34 ± 1.89 vs. 0.58 ± 0.58 changes of direction per min; Wyon et al., 2011). Additionally, classical ballet is more intermittent, consisting of periods of higher intensity activity, and longer durations of rest, subsequently incurring a significant anaerobic stress (Wyon et al., 2011). The perception of effort has previously been shown to be elevated during intermittent exercise compared with steady-state exercise of an equivalent internal load (Drust et al., 2000; Cerda-Kohler et al., 2015). The stronger relationships observed in the present study compared with previous research in contemporary dancers, would therefore not appear to be a result of the difference in genre. An alternative explanation would be that the very large relationships we report could be explained, in part, by the difference in training history between cohorts. In support of this, research in swimmers (Barroso et al., 2014), and athletes of mixed experience (Winborn et al., 1988) revealed that the validity of s-RPE is proportional to athletic experience.

Consistent with both Jeffries et al. (2016) and Surgenor and Wyon (2019), we report weaker relationships between s-RPE and objective measures in ballet class compared with rehearsals. While each of these studies attributed this finding to the difference in dance genre (i.e., ballet class vs contemporary rehearsals), and ballet not being the participants’ primary discipline, neither of these explanations explain the current results. One explanation may be that the cohort’s familiarity with the structure, environment, and teachers of ballet class, compared with the more changeable nature of rehearsals, may have mediated this relationship. Factors such as the psychological demands, the individuals present, and the external environment of a session, for example, have all been proposed as influences on the relationship between physiological and perceptual stress (Haddad et al., 2017). We suggest, however, that this finding is most likely be a result of little inter-session variation; class follows a consistent structure each day, progressing in both physical and technical complexity from barre, to center, and finally allegro. Differences in perceived exertion are therefore finer and harder to distinguish on a 10-point scale. Additionally, this results in little variation in TL, as the duration and content of ballet class do not allow the dancer to reach particularly small or large TLs, to which Pearson correlation is sensitive (i.e., range restriction; Goodwin and Leech, 2006). The lack of change in rmcorr when analyzing rehearsals alone vs. rehearsals and ballet class (where the range restriction is not present), supports this idea. Practically speaking, s-RPE is therefore only less valid when attempting to distinguish between a set of relatively homogeneous sessions.

While male and female classical ballet dancers jump, plié, and change direction at similar rates, they differ in their requirement to lift (Wyon et al., 2011) and dance en pointe, respectively. During lifts of their partners, male dancers undertake L5/S1 compression forces in excess of 4000 N, and shear forces in excess of 500 N (Alderson et al., 2009). In this regard, elements of male roles bear a resemblance to resistance exercise, and could therefore be expected to result in differing perceptions of effort (Sweet et al., 2004). Pointe work on the other hand, is incomparable to any other exercise modality; given the large pressures on the first and second toes, the perceived effort may be inconsistent with the dancer’s HR (Salzano et al., 2019). Additionally, male and female intensity profiles differ, with females spending a larger duration performing at moderate and hard exercise intensities, and males performing very high intensity multi jump routines closer resembling intermittent exercise (Wyon et al., 2011). Despite these differences, we report similar relationships between s-RPE and objective measures in both males and females. The s-RPE method is therefore a valid tool for monitoring internal TL, regardless of sex.

### Limitations

Given the professional level of the current cohort, it was not possible to formally measure each participant’s maximum HR. It is important to note that this will have resulted in less accurate measurements of objective measures of internal TL. Additionally, as the current cohort are considered elite ballet dancers, practitioners should consider that s-RPE may not demonstrate the same degree of validity in non-elite populations. Due to the novelty of this type of testing within the cohort, participants were given a large degree of control over the sessions in which data were collected. While we report measures of intensity and duration for the sessions completed within the current study, these data should therefore not be considered normative values for ballet class and rehearsal. The relatively small total number of sessions completed by female dancers, as well as the total number of female dancers involved must be considered when interpreting results regarding differences in sex. Finally, although s-RPE data are ordinal in nature, in the present analysis we use parametric tests which treat them as continuous data.

### Practical Applications and Further Research

Unlike traditional measures used to assess internal TL (e.g., HR, oxygen uptake, and blood lactate concentration), the s-RPE method is a cost-effective and non-invasive means of monitoring internal TL. The high volumes of rehearsal undertaken by classical ballet dancers are well documented (Cohen et al., 1980; Allen et al., 2012), and have been linked with maladaptive responses leading to increased risk of injury and overtraining. The current results provide a means by which the daily and weekly TLs of dancers may be used to implement periodization models with the aim of optimizing health and performance. Differential s-RPEs have been used in sporting environments to understand multiple types of physical exertion, and the training stress imposed on multiple body parts (McLaren et al., 2017). Given the large number of different physical stressors involved in classical ballet (e.g., pointe work, jumping, and lifting, etc.), the use of differential s-RPEs may provide additional insight into the TLs undertaken by dancers. Finally, understanding the validity of s-RPE is important for non-professional institutions (e.g., ballet schools) who may not have access to alternative measures of TL; further research is therefore warranted into the validity of s-RPE in these populations.

## Conclusion

This study investigates the convergent validity of s-RPE with two objective measures of internal TL in professional classical ballet dancers. We demonstrate very large rmcorr between s-RPE and Edwards and Banister TRIMPs, as well as intra-individual relationships ranging from moderate to almost perfect. Sub-analyses revealed that correlation coefficients were similar between male and female participants, however, relationships were stronger in rehearsals compared with ballet class. These results are similar to findings previously reported in both sport and dance research, and support the use of the s-RPE method as a valid and practical tool for measuring internal TL in professional classical ballet dancers.

## Data Availability Statement

The raw data supporting the conclusions of this article will be made available by the authors, without undue reservation, to any qualified researcher.

## Ethics Statement

The studies involving human participants were reviewed and approved by St Mary’s University, Twickenham. The patients/participants provided their written informed consent to participate in this study.

## Author Contributions

All authors contributed to the conception and design of the work. JS completed the acquisition, analysis, and interpretation of the data for the work. All authors drafted the work or revised it critically for important intellectual content, approved the final version of the manuscript, and agreed to be accountable for all aspects of the work ensuring accuracy and integrity.

## Conflict of Interest

The authors declare that the research was conducted in the absence of any commercial or financial relationships that could be construed as a potential conflict of interest.
